# Small-area socioeconomic deprivation indices in Cyprus: development and association with premature mortality

**DOI:** 10.1186/s12889-019-6973-0

**Published:** 2019-05-22

**Authors:** Demetris Lamnisos, Galatia Lambrianidou, Nicos Middleton

**Affiliations:** 1 Department of Health Sciences, School of Sciences, European University Cyprus, 6, Diogenes Str. Engomi, P.O.Box 22006, 1516 Nicosia, Cyprus; 2 Department of Nursing, School of Health Sciences, Cyprus University of Technology, Limassol, Cyprus

**Keywords:** Socioeconomic deprivation index, Social inequalities in health, Mortality, Small-area analysis, Principal component analysis, Poisson spatial model, Ecological study, Cyprus

## Abstract

**Background:**

Area-level measures of socioeconomic deprivation are important for understanding and describing health inequalities. The aim of this study was the development and validation of a small-area index of socioeconomic deprivation for Cypriot communities and the investigation of its association with the spatial distribution of all-cause premature adult mortality.

**Methods:**

Six area-level socioeconomic indicators were used from the 2011 national population census (low educational attainment, unemployment, not owner occupied household, single-person household, divorced or widowed and single-parent households). After normalization and standardization of the geographically smoothed indicators, Principal Component Analysis (PCA) was used to construct indicator weights. The association between deprivation indices and the spatial distribution of all-cause premature adult mortality was estimated in Poisson log-linear spatial models.

**Results:**

PCA resulted in two principal components explaining the 65.7% of the total variance. The first principal component included four indicators (low educational attainment, single-person households, divorced or widowed and single-parent households, the latter however with a negative loading) and it thought more likely to capture rural-related aspects of deprivation. The second principal component included the other two indicators (unemployment and not owner occupied households) and it is more likely to capture urban-related aspects of material deprivation. Restricting the analysis in the metropolitan areas of the island resulted in a different set of indicators for the urban-specific deprivation index. All developed indices were linearly associated with all-cause premature adult mortality. The all-cause premature adult mortality increased by 17% per 1 standard deviation (SD) increase in rural-related socioeconomic deprivation (95% CrI: 8–27%) and 8% per 1 SD increase in urban-related aspects of material deprivation (95% CrI: 3–15%) in the nationwide analysis and 9% per 1 SD increase in urban-specific socioeconomic deprivation (95% CrI: 4–15%) across metropolitan areas.

**Conclusions:**

The results of this study demonstrate that a set of small-area indices of socioeconomic deprivation across Cypriot communities have good construct and predictive validity. However, the study indicates that different aspects of socioeconomic deprivation may be important in rural and urban areas in Cyprus. The developed socioeconomic deprivation indices could offer a valid new tool for Cypriot public health research and policy in terms of identifying areas in greatest need, guiding resource allocation and developing area-targeted public health programmes and policies.

**Electronic supplementary material:**

The online version of this article (10.1186/s12889-019-6973-0) contains supplementary material, which is available to authorized users.

## Background

Socioeconomic deprivation is a multidimensional concept as it refers to the relative disadvantage an individual or a social group experiences (including a group defined in geographical terms e.g. a community or a neighborhood) in terms of access and control over economic, material or social resources and opportunities. Thus, it is a complex construct, difficult to operationalize since it involves many aspects that pertain to the economic and material domain, such as employment, income, education, occupation, housing, as well as the social domain, such as social position, family support and social integration. At the level of the individual, either the classic triad of education, occupation and income and/or subjective measures of an individual’s own perceived position in the social ladder, are used to operationalize a person’s socioeconomic status [1], while composite indices of relative deprivation have also been described [2]. One example is DiPCare-Q, a 16-item screening tool for primary care scale tapping on material aspects of deprivation e.g. difficulty paying bills, ability to buy food or clothes, etc. and social deprivation e.g. no holidays, no evenings with friends etc. [3]. When geographically defined, the concept is commonly operationalized using single and compound indicators to measure relative deprivation across defined area units, such as census tracts, at city, district or national level. Compound indicators are constructed using a combination of various single area-level indicators, in order to consider multiple dimensions of deprivation [4–12]. Commonly, predictive validity of such compound indices is assessed in terms of the observed social gradient in all-cause or cause-specific mortality or premature mortality (commonly before the age of 65), or other health outcome. A different promising approach of validating area-based socio-economic indices has been described in the construction of the European Deprivation Index (EDI) for small areas across five European countries, England, France, Italy, Portugal and Spain [13, 14]. The EDI was developed using aggregated data from the 2001 census and individual data from the 2006 EU-SILC survey (European Union-Statistics on Income and Living Conditions). The EDI was created from a weighed sum of census indicators associated with an individual deprivation indicator and the weights assigned to those indicators were resulted from a multivariable regression model.

Area-level socioeconomic deprivation indices are commonly used in public health research to either quantify the magnitude of geographically-determined social inequalities in health or to assess the potentially independent effect the characteristics of these areas have on health beyond that of the individual socioeconomic position [15, 16]. Indices of area-level socioeconomic deprivation are also useful to substitute missing individual-level data in epidemiological studies, for example, in the context of adjusting for confounding for socioeconomic factors. Moreover, government public health authorities commonly use these indices to facilitate the identification of areas in greatest need, guide resource allocation and target policies and programmes to geographic areas where health care needs and demand is expected to be highest.

In Cyprus, evidence of the social patterning of health is very limited and one may have to fit together the pieces of the puzzle through a series of fragmented findings from studies whose main focus is not to explicitly explore the social gradient in health. Recognizing that social stratification is also geographically defined, area-based measures place a community in the socioeconomic disadvantage continuum and are used to quantify the magnitude of geographically defined social inequalities in health. However, there are no commonly accepted area-level socioeconomic deprivation indices, or even single indicators for that matter. The importance of socioeconomic indicators in monitoring systems is best expressed by Marmot et al. (2008): “Experience shows that countries without basic data on mortality and morbidity stratified by socioeconomic indicators have difficulties in moving forward on health equity” [17]. A “borrowed” Townsend-like index (“unemployed economically active population”, “not owner occupied households”, “households with >1 person/room” and replacing “households with no car”, which is not relevant in a Cypriot context with “households with no access to a personal computer”) was considered using 2001 and subsequently 2011 census data but did not perform well in the Cypriot context [18–20]. The internal consistency between the four components was insufficient as indicated by the Cronbach’s alpha value of 0.36. Furthermore, the results of a spatial hierarchical model for factor analysis of the four components revealed that the shared component was driven by only one of the indicators, specifically the “households with no access to a PC”. The percentage of the total variability explained by the shared component for two indicators – unemployed economically active population and not owner occupied households – was negligible (less than 1%). Not only the components did not share a common geography, but also the Townsend-like index was not associated with premature mortality [20].

Validating a deprivation index means verifying whether it adequately reflects the reality being measured and this is a complex exercise because the index must respond to a number of criteria and have certain properties [21]. Previous deprivation indices were validated using a combination of the construct and criterion validation methods. Construct validity of the socio-economic deprivation indices was most frequently evaluated by Principal Component Analysis (PCA) or Factor Analysis [4, 5, 7, 10, 22–24] while criterion validity is most commonly established by comparing the area-based variation of new indices to indices already in use, such as the Townsend deprivation index [4, 6, 8], which nevertheless was not found to be adequate in a Cypriot context. Predictive validity of a new index has been typically assessed by examining its association with all-cause and cause-specific overall or premature mortality, as it would be expected to capture a social gradient [6, 7, 9, 10, 21, 23, 25, 26].

Therefore, the objective of this study was to investigate the construct and predictive validity of a country-specific deprivation index across Cypriot municipalities and communities. A defining characteristic of Cyprus is the striking distinction between the ageing and resource-scarce rural communities Vs younger more educated populations in metropolitan areas. Since a single deprivation index may not discriminate between rural and urban-specific dimensions of deprivation [27, 28], we also sought to assess whether a different set of indicators are more relevant across rural and metropolitan areas of the island.

## Methods

### Data sources and data

Demographic and socioeconomic data were obtained from the 2011 national population census, conducted by the National Statistical Service. Individual data were aggregated at the level of geographical units used for census purposes. There are *N* = 369 areal units in the territory controlled by the Republic of Cyprus, termed municipalities, in Greek ‘Demoi’ in urban and communities, ‘Koinotites’, in rural areas. These geographical areas are the smallest geographical units for which both census and mortality data are available. The upper geographic unit of aggregation is the “District” level, which is too large (*N* = 5 districts).

Six census-based area-level indicators were selected on the basis of an original literature review of similar studies which suggests that the most commonly represented indicators across European socio-economic deprivation indices are measures of educational attainment, unemployment, house tenure, and household amenities. Furthermore, commonly, sociodemographic groups “at risk”, such as pensioners and single parents, are often represented in these indices. To be selected, indicators needed to meet three criteria: previous use in the construction of deprivation indices, affinity with the material and/or social dimensions of deprivation and availability at the municipality/community level [4, 6–10, 13, 23, 25, 26, 29, 30]. It is also of note that a previous data-driven exercise among a wider set of 26 indicators from 2001 census suggested that these are the indicators which were most strongly associated with premature mortality in an earlier time period [31]. These indicators were the proportion of people with low educational attainment (defined as Number of adult people with at most lower secondary level education/Total population over 15 years of age), the proportion of unemployed economically active population (Number of unemployed people/Total economically active population), the proportion of non-owner occupied households (Number of not owned households/Total households in conventional dwellings), the proportion of single-person households (Number of households with a single person/ Total number of households), the proportion of divorced or widowed population (Number of divorced or widowed people/Total population) and the proportion of single-parent households (Number of single-parent households/Total number of households). Only two of these indicators (unemployment and not-owner occupied households) are components of the Townsend index of material deprivation. All census indicators were publically available except the proportion of divorced or widowed population which was obtained after request from the Cyprus Statistical Service.

The observed number of gender- and age group-specific (in 5-year age bands from 15–20 to 60–65) all-cause premature adult mortality in the period 2009–2011 for each community was obtained after permission from the Health Monitoring Unit of the Cyprus Ministry of Health. Indirect standardization was used to calculate Standardized Mortality Ratios (SMRs), by applying the national gender and age-specific mortality rates on the gender and age-specific population sizes of each community to calculate the expected number of all-cause mortality for each community.

Finally, a composite measure to place communities in an urban-rural continuum defined as the sum of z-score values of population density - a typical measure of rurality, and population potential – a measure of remoteness from large centres of population. Population density was calculated as the number of people per square kilometer and population potential was calculated for each community as the sum of the ratios of population at all other communities to the distances from the community in question to all the other communities. It is of note that based on European-wide thresholds, around 5% of areas would be classified as urban in Cyprus. Furthermore, in contrast to a categorical classification of urbanity, a continuous variable combining the population density and population potential also distinguishes between areas of low population density (otherwise classified as rural) depending on their proximity to metropolitan areas.

### Statistical methods

All indicators apart from the proportion of people with “low educational attainment” were first transformed using the logarithmic transformation log(1 + *x*) in order to normalize their distribution. For each indicator, the extent to which adjacent geographical areas display similar socio-economic characteristics was first assessed. As indicated by the percentage of spatially structured variability to the total variability, all indicators exhibited spatial autocorrelation which ranged from moderate to quite marked (e.g. 73.5% for educational attainment). Furthermore, as expected there was lower precision in less dense rural and remote communities with small underlying population denominators. Therefore, a univariate Gaussian spatial model was used to incorporate spatial dependence and to provide smooth estimates of each census indicator. This model assumes that the value of a census indicator in any area is the sum of three terms, one constant term (the national mean) and two random terms. The first random term includes an unstructured normally distributed error to account for the global between-area variability (i.e. the variability in the values of census indicator across all areas). The other random term is a spatially structured random effect accounting for the local variability and capturing the tendency of neighboring areas to exhibit similar levels in the specific socio-economic characteristic under study. The spatially structured random effect was assumed to follow the Conditional AutoRegressive (CAR) model [32] with first order adjacency to define a community’s neighbours (i.e. those communities sharing a common border). This univariate Gaussian spatial model resulted in smoothed estimated values of a census indicator in a given area that was a form of a weighted average between the raw value and the average value of neighboring areas with weights depending on the population size of the area.

The Pearson correlation coefficient was used to investigate the association between the normally distributed indicators while the non-parametric Spearman correlation coefficient was used for the non-normally distributed indicators. Internal consistency of a unidimensional deprivation index was measured with Cronbach’s alpha, which evaluates the extent to which a set of indicators represents a single latent construct. A Cronbach’s alpha value greater than 0.7 indicates a sufficient internal consistency. The construct validity of the socio-economic deprivation index was investigated through the Principal Component Analysis (PCA) with varimax rotation and Kaizer normalization of the components with eigenvalue greater than 1, after assessing its suitability by the Kaiser-Meyer-Olkin (KMO) coefficient and Bartlett test of sphericity. KMO coefficient greater than 0.6 and statistically significant Bartlett test of sphericity are suggesting that the data are suited for PCA [33]. PCA is a widely used multivariate technique for calculating indices of deprivation [4, 5, 7, 10, 34]. The objective is to account for the uncertainty of a set of indicators with the fewest components possible leading to dimension reduction. These components are constructed as linear weighted combinations of the original indicators and allows the best contrast between areas. The PCA determines the weights for each of the indicators in each component [35]. PCA was performed in two different cases, the first PCA was performed to all areal units (*N* = 369 area units) to construct the nationwide deprivation indices and the second PCA was performed to metropolitan areas only (*N* = 119 areal units) to develop the urban-specific deprivation indices.

The predictive validity of the socioeconomic deprivation indices was assessed in terms of their ability to capture a social gradient in all-cause premature adult mortality. Poisson log-linear spatial models were used to investigate the observed association between the proposed index and mortality. The dependent variable of this model was the observed number of all-cause premature adult mortality (ages 15–65), the offset term was the expected number of all-cause premature adult mortality and the spatial random effect follows the CAR model with first-order adjacency [32]. The independent variable was the socioeconomic deprivation index introduced in the model either as a z-score variable or as a categorical variable representing quartiles of areas with increasing levels of socio-economic deprivation (Q1-least deprived to Q4-most deprived) in order to facilitate interpretation. Concurrent criterion-validity was not considered since composite indices of deprivation are not traditionally used in Cyprus, and there are not any commonly accepted area-level socioeconomic indices (composite or single indicators) used in local research or in policy. In fact, this is the first study addressing the geographical patterning of socio-ecomomic characteristics of Cypriot communities.

The univariate Gaussian spatial model and the Poisson log-linear spatial model were implemented in a Bayesian setting which requires a specification of the prior distributions for the regression coefficients and the variance components. All statistical analyses were performed in SPSS (version 20) except the univariate Gaussian spatial model and Poisson log-linear spatial model that were performed in the statistical software R [36] and WinBUGS [37]. Significance tests and confidence intervals were calculated at a significance level of 5% for the frequentist methods while 95% credibility intervals were computed for the spatial models.

## Results

The median population size across 369 Cypriot communities (mean land area in square kilometer 15.41 km^2^) was 316 people, IQR: 110.50–1123.50, while only 10% of areas had a population of over 4100. Due to the small size of the island, these communities are particularly small by comparison to similar studies in the literature and the mean population size (2277.5 inhabitants) does not provide a characteristic measure. In half of the 369 Cypriot communities the percentage of adult population with at most lower secondary education was below 47.7% (IQR: 37.2–60.2), the percentage of unemployed economically active population was below 10.1% (IQR: 7.1–13.1), the percentage of not owner occupied households was below 21.4% (IQR: 10.4–36.0), the percentage of single-person households was below 19.3% (IQR: 14.8–26.2), the percentage of divorced or widowed was below 9.1% (6.7–13.7) and the percentage of single-parent households was below 5.5 (IQR: 3.6–7.2) (Table 1).

**Table 1:** Median and interquartile range (IQR) of the population size and the socio-economic indicators across 369 communities of Cyprus

Indicator	Median (IQR)
Population (*N*)	316.0 (110.5–1123.5)
At most lower secondary education (%)	47.7 (37.2–60.2)
Unemployed economically active (%)	10.1 (7.1–13.1)
Not owner occupied households (%)	21.4 (10.4–36.0)
Single-person households (%)	19.3 (14.8–26.2)
Divorced or widowed population (%) Single-parent households (%)	9.1 (6.7–13.7)
	5.5 (3.6–7.2)

Generally, pairwise correlations among the geographically-smoothed indicators revealed a mixed pattern (Table 2). For example, in terms of low educational attainment, there was a positive correlation with single-person households and divorced/widowed population, yet, a negative correlation with unemployment and single-parent households and no correlation with not-owner occupied households. There was only moderate positive correlations between some indicators, in the range of 0.5–0.7. The highest correlation was observed between proportion of divorced or widowed population and single-person households (r = 0.78). Unemployment and house ownership, the two indicators included in the Townsend index, only had a small positive correlation between them (r = 0.23) and appeared to be poorly correlated with the rest of the variables.

**Table 2:** Bivariate correlations between socioeconomic indicators

Indicator	At most lower secondary education	Unemployed population	Not owner occupied households	Single-person households	Divorced/ Widowed population	Single-parent households
At most lower secondary education	1	−0.22*	−0.03	0.48*	0.58*	−0.47*
Unemployed economically active population		1	0.23*	0.04	0.15*	−0.09
Not owner occupied households			1	0.23*	0.05	0.00
Single-person households				1	0.78*	−0.60*
Divorced or widowed population					1	−0.63*
Single-parent households						1

*p-value < 0.05

The Kaizer-Meyer-Olkin coefficient was estimated at 0.67, while the Bartlett test of sphericity was 680.9 (*p* < 0.001). Table 3 presents the results of PCA to all areal units (*N* = 369). Two principal components with eigenvalue greater than one were rotated and explained the 65.7% of the total variance. The first principal component includes four of the socio-economic indicators: population with low educational attainment, single-person households, divorced or widowed population and single-parent households, the latter however with a negative loading. Internal consistency of the scale was sufficient since the Cronbach’s alpha coefficient was 0.83. Computation of partial (if item deleted) coefficients showed that each item is important for the scale. The first principal component was strongly correlated (r = 0.83) with the composite indicator of rurality and remoteness and it is thought to reflect rural-related aspects of deprivation in the Cypriot context. This principal component was named “Rural-related SE deprivation”. The second principal component included the other two indicators, unemployed economically active population and not owner occupied households, explaining 21.8% of the total variance, and though to tap on aspects of material deprivation. Since home ownership is higher in rural areas of Cyprus and unemployment is lower, this component is more likely to capture urban-related aspects of material deprivation and it was termed “Urban-related aspects of material deprivation”.

**Table 3:** Rotated factor pattern from principal component analysis of indicators

Indicator	Factor I	Factor II	Communalities
Population with at most lower secondary education	0.74		0.64
Unemployed economically active population		0.78	0.61
Not owner occupied households		0.73	0.54
Single-person households	0.85		0.77
Divorced or widowed population	0.87		0.78
Single-parent households	−0.77		0.60

Extraction method: Principal component analysis, rotation method: Varimax with Kaizer normalization. Loadings with an absolute value of ≥0.40 are displayed

Table 4 presents the results of PCA to metropolitan areas only (*N* = 119 area units). The indicator single-person households cross-loaded on two principal components and was removed. The first principal component of the remaining five indicators loaded on all indicators except “population with at most lower secondary education” and explained 40.9% of the total variance. Internal consistency of the scale was sufficient since the Cronbach’s alpha coefficient was 0.72. Computation of partial (if item deleted) coefficients showed that each item is important for the scale. This principal component was named “Urban-specific SE deprivation”. The second component loaded only on the remaining one indicator “population with at most lower secondary education”, and was thus not taken further. Different factor analysis and rotation methods were applied but the construction of the deprivation indices remained the same.

**Table 4:** Rotated factor pattern from principal component analysis of indicators for the metropolitan areas (*N* = 119)

Indicator	Factor I	Factor II	Communalities
Population with at most lower secondary education		0.94	0.89
Unemployed economically active population	0.55		0.45
Not owner occupied households	0.73		0.53
Divorced or widowed population	0.79		0.63
Single-parent households	0.76		0.60

Extraction method: Principal component analysis, rotation method: Varimax with Kaizer normalization. Loadings with an absolute value of ≥0.40 are displayed

Summary measures of the census indicators for each deprivation index and quartile are presented in Additional file 1. Table 5 shows Relative Risks (RR) of all-cause premature adult mortality per one standard deviation (SD) increase and across quartiles of areas with increasing levels of deprivation in a national level analysis (*N* = 369) and restricted to metropolitan areas (*N* = 119). There was a stepwise increase across increasing levels of rural-related socioeconomic deprivation in the nationwide analysis. Premature mortality appeared 30% higher in the most deprived (and rural) areas (95% CrI of RR: 0.96, 1.74), even though the credible interval is wide due to the particularly small size of the population in rural areas. Communities in the third quartile of deprivation had 33% higher mortality (95% CrI of RR: 1.05, 1.64) as compared with the least deprived communities while communities in the second quartile did not have significantly higher mortality. Similarly, there was a stepwise increase across increasing levels of urban-aspects of material deprivation, with 33% higher mortality rates in the most deprived areas (95% CrI of RR: 1.11, 1.63). While higher mortality rates were observed in communities classified in the second and third quartiles of deprivation, as compared with the least deprived communities, these estimates were not statistically significant. Finally, in metropolitan areas, the urban-specific socioeconomic deprivation index also captured a stepwise association in premature adult mortality, with 36% higher rates in the most deprived urban areas in comparison to the least deprived urban areas (95% CrI of RR: 1.15, 1.62). Estimates of mortality in the middle two quartiles, while progressively higher than the least deprived quartile, were not statistically significant.

**Table 5:** Relative Risk (and 95% Credible Intervals) of premature adult mortality per one standard deviation (SD) increase and across quartiles of PCA factors

	Per 1 SD increase	Q1-least deprived	Q2	Q3	Q4-most deprived
Rural-related SE deprivation (*N* = 369)	1.17 (1.08, 1.27)	Ref	1.07 (0.94, 1.23)	1.33 (1.05, 1.64)	1.30 (0.96, 1.74)
Urban-aspects of material deprivation (*N* = 369)	1.08 (1.03, 1.15)	Ref	1.16 (0.96, 1.42)	1.16 (0.96, 1.43)	1.33 (1.11, 1.63)
Urban-specific SE deprivation (*N* = 119)	1.09 (1.04, 1.15)	Ref	1.09 (0.90, 1.31)	1.13 (0.95, 1.37)	1.36 (1.15, 1.62)

## Discussion

The objective of this study was the development and validation of Cypriot small-area indices of socioeconomic deprivation and the investigation of their association with all-cause premature adult mortality. PCA was used for the construction of the deprivation indices. At a nationwide level (*N* = 369 municipalities/communities), PCA resulted in two principal components explaining the 65.7% of the total variance. The first principal component included four indicators (low educational attainment, single-person households, divorced or widowed and single-parent households, the latter however with a negative loading) and it was tapping on aspects of deprivation that are by default more prevalent in rural areas in Cyprus, thus, making it hard to delineate the effects of deprivation from rurality itself. This principal component was named “Rural-related socioeconomic deprivation”. The second principal component included the other two indicators (unemployment and not owner occupied households) and it appeared to contrast urban versus rural communities in terms of material aspects of deprivation. Therefore, this principal component was termed “Urban aspects of material deprivation”. Restricted analysis for the metropolitan areas only (*N* = 119 municipalities/communities) resulted in the “Urban-specific socioeconomic deprivation”. This index was based on a slightly different set of variables, which now include both material aspects (unemployment and not owner occupied households) and social aspects of deprivation (divorce/widowed and single-parent households). All developed indices were linearly associated with all-cause premature adult mortality indicating their predictive validity in revealing the social gradient in health. Although Cyprus is a rather small country, there was a gradient in the risk of mortality according to the socioeconomic characteristics of the areas. Similar consistent patterns of socioeconomic inequalities in mortality were reported across 16 European cities [35].

It is perhaps not surprising that PCA analysis at the national level tapped on two aspects of deprivation – social and material – but at the same time, revealed the differed nature of deprivation between urban and rural areas of the island, as different aspects of deprivation may be more relevant in rural versus urban areas. The fact that a single index cannot always discriminate well between rural-related and urban-specific aspects of deprivation has been discussed before in the literature [27, 38]. In fact, one criticism of most deprivation indices is their focus on urban aspects of deprivation. Often, the indicators used to construct these indices have a different meaning in urban and rural areas. To give an example, car ownership which is typically included in deprivation indices [39, 40] may not be an indicator of material wealth in rural areas where having a car is a necessity [38, 41]. This urban view of deprivation may explain the weaker association with health indicators observed in rural areas as they fail to capture rural-related aspects of deprivation [27, 38, 42–44]. Similarly, in this case, it seems that rural areas in Cyprus are generally more disadvantaged compared to urban areas in terms of social aspects, which according to Townsend refers to rights in “family activities, integration into the community, formal participation in social institutions, recreation and education”. This does not go to say that material disadvantage is not relevant is rural areas, since the findings might also suggest that material aspects of deprivation are not fully captured, for instance ‘hidden’ seasonal unemployment not recorded in official statistics. Therefore, since socio-economic indicators may have different meaning in rural and urban areas, it seems reasonable to use different deprivation indices in a nationwide analysis and in an urban context.

The six indicators used to construct the deprivation indices were selected from the available census indicators at the municipality/community level and consistently with previous literature for the construction of deprivation indices [4, 6, 8–10, 13, 23, 25, 26, 29, 30]. While the pre-selected indicators are thought to tap on different aspects of material or social deprivation (family status, education, employment and housing tenure), it should be noted that the distinction between material and social is not as clear, and it is generally considered that social deprivation is harder to measure [45]. A single indicator may be thought to tap on both material and social aspects of deprivation. For example, unemployment (a component of the Townsend index of material deprivation) is traditionally considered to reflect material deprivation since it is a condition or state associated with lack of resources and amenities (for example, in many of the traditional UK-based deprivation indices, such as the Townsend and the Jarman indices). In contrast, in Forrest and Gordon’s (1993) MatDep and SocDep indices (referring to material and social deprivation respectively), unemployment is included in the social deprivation index along with single-person and single-parent households, conceived as an “indirect indicator” referring to the victims to deprivation, rather than a “direct indicator” of lack of resources and amenities (for instance, no access to a car). In fact, as suggested in a recent review of the literature, newly developed indices across Europe do not attempt to make a distinction between material and social deprivation [31].

Our review also suggests that while traditionally UK-based indices were commonly “borrowed” and assumed to be cross-nationally valid, in recent years there has been a growing interest in developing home-grown indices across Europe. The number, and more importantly the population size of areas, vary widely across studies – from quite large areas (and hence possibly heterogeneous in terms of the socioeconomic environment of the various smaller areal units/neighborhoods that comprise them) to tens of thousands of areas with much smaller populations. The majority of constructed indices are based on census data, or other routine sources of data and commonly used between four to ten pre-selected variables based on previous use and/or data availability, and less so on theoretical grounds [31]. It seems that some measure of educational attainment, unemployment and occupational-based social class are the indicators most commonly represented across these indices, while income measures are quite rare, commonly due to data unavailability as was the case in this study. Sociodemographic groups “at risk” of deprivation on the other hand, such as single-parent households and single-person households, are also quite common across these indices, while many also include some measure of house tenure, like the case of this study. Car ownership or other amenities, for example access to a PC, are also common; however, they were not thought as relevant in the Cypriot context since the first is not recorded in the census and the latter did not seem to perform adequately [18]. In some cases, an exploratory approach is taken whereby PCA is used for variable selection from a larger set, which intentionally includes redundant variables, for example as many as 48 [8] and 52 [4]. While the six variables tap on aspects of both material and social deprivation, the fact that the current study focused on a small set of pre-selected variables on the basis of the literature is a limitation. Nevertheless, this is the first study to explore the extent to which area-level socio-economic characteristics capture the social gradient in mortality in Cyprus. Other than the limited availability of data in the Cypriot census, an advantage of a small number of indicators is the avoidance of several redundant variables that may belong to the same domain leading to inflated loadings of principal components. A small number of indicators in the composition of a deprivation index provides parsimony, simplifies comprehension, especially among policy-makers not familiar with these approaches, while it is more likely to allow comparability across time.

A different promising approach of constructing and validating area-based socio-economic indices has been described in the construction of the European Deprivation Index (EDI) for small areas across five European countries, England, France, Italy, Portugal and Spain [13, 14]. Other than the cross-national adaptability of the approach, the main strength is the theoretical formulation of deprivation as “unmet need” due to lack of resources considered customary in a given societal context. Using data from the EU-Statistics on Income and Living Conditions Survey, the method involved identifying needs considered fundamental in each country defined as those not lacking in more than 50% of households and associated with objective (i.e., actual income level) and subjective (i.e., self-reported financial difficulties) poverty. For instance, while the ability to cover unplanned expenses was identified as a fundamental need across all five countries, not having a car was not a fundamental need in France or Spain. At the second stage, a weighted combination of census-based indicators more predictive of areas with a higher proportion of households lacking two or more fundamental needs was selected. An 8–10 variable index was constructed for each country, with only four indicators common to all.

The proposed deprivation indices have limitations shared by most of the deprivation indices. The developed indices are based on decennial census data, and thus may not reflect changes in the socio-economic environment in the period between the national censuses. Furthermore, they are calculated for administrative units as used of the census. While in general, the population size of these areas is smaller than similar studies in the literature due to the particularly small size of Cyprus, they also include a number of large areas (municipalities), which are possibly heterogeneous in terms of the socioeconomic environment of the various smaller area units (neighbourhoods) that comprise them [46]. The advantages that come from working with areas of relatively equal population size or social homogeneity were emphasized in [47] as the size and configuration of areal units can directly influence the results of any statistical analysis and this problem is known as the modifiable area unit problem [48]. Moreover, because of its ecological design, our study presents the typical limitations of all such studies known as the ecological fallacy. The developed indices are aggregated measures of deprivation and therefore the relations observed between the variables at the geographical level cannot be directly applied to the individual level [49].

A two-step process was applied to construct the socioeconomic deprivation indices in Cyprus. In the first step, a univariate spatial model that account for the underlying spatial structure of census data was used to increase precision for estimated values of indicators. This spatial smoothing of the indicators was deemed necessary because the underlying population denominators are small for less dense areas, which nevertheless represent the vast majority of areal units in the analysis (25% of the communities have a population less than 110 inhabitants) and this results in high uncertainty (low precision) in the estimates of the indicators for those areas. A potential drawback of spatial smoothing is the introduction of spatial homogeneity between adjacent areas and this may reduce the index’s capacity to distinguish communities. In the second step, a PCA was used for the construction of deprivation indices. PCA is commonly used to combine socioeconomic indicators into a composite index in order to place communities in the socioeconomic deprivation continuum [4–8, 10]. Although PCA is helpful in revealing underlying dimensions of the latent construct of socioeconomic deprivation, it assumes that all areas are homogeneous in possessing these area dimensions. As such, they ignore the possibility that there might exist a diversity of areas with different combinations of these dimensions [50]. Furthermore, aggregated measures from PCA cannot identify the distinct characteristics, or combination of characteristics, attributable to specific geographical areas which may explain the observed variations in population health [51].

The exploratory nature of this study indicates that different aspects of socioeconomic deprivation may be important in rural and urban areas in Cyprus. This challenge in developing a deprivation index has also been observed in other studies [5, 9] and researchers should consider this aspect in developing deprivation measures that cover rural areas [9]. This issue has been highlighted in the UK [38, 41] and in developing the Scottish Index of Multiple Deprivation [52], but appropriate solutions are still being developed and may vary across countries. Therefore, future efforts should concentrate in developing urban and rural-specific indices of socioeconomic deprivation, perhaps by using a wider set of indicators. A promising procedure in selecting the common determinants of socioeconomic deprivation in different areas is the one presented in [8]. They proposed a statistical procedure which is based on a successive use of principal component analysis to select indicators among a given set that are common determinants of socioeconomic deprivation at a national level and specific to urban and rural areas. This statistical procedure will provide a separate set of socioeconomic indicators for rural and urban areas and will allow the construction of rural and urban specific indices of socioeconomic deprivation. However, rural and urban specific indices of socioeconomic deprivation have the disadvantage of restricting comparisons between geographical areas at a national level. In any future study on the construction of deprivation indices, it may also be warranted to age-standardize some indicators as their meaning varies for different birth cohorts [5]. For instance, it is expected that older cohorts will be over-represented in those classified as having lower education. Thus, census data on educational attainment as well as other routinely available indicators with a clear age pattern should be stratified by age-group in official statistics.

## Conclusions

There are currently no area-based indices of deprivation in Cyprus and this is the first geographical study to explore the variability in socioeconomic disadvantage across Cypriot communities. The development of area-level socioeconomic deprivation indices is more relevant now than ever, since the country is undergoing the largest national health care system reform, including the introduction of General Practitioners. The developed socioeconomic deprivation indices will allow government public health authorities to facilitate the identification of areas in greatest need, guide resource allocation of health and other investments, target prevention strategies and plan local health promotion programmes aimed at reducing geographical health inequalities. Furthermore, they will allow further research into the magnitude of geographical health inequalities and the potential health effects of the socioeconomic characteristics of a place over and above individual socioeconomic characteristics.

## Additional files

Additional file 1: Median values of the census indicators for each deprivation index and quartile. Summary measures of the census indicators for each deprivation index and quartile (DOCX 12 kb)

## Abbreviations

CAR: Conditional AutoRegressive
CrI: Credible Intervals
EDI: European Deprivation Index
IQR: Interquartile range
PCA: Principal Component Analysis
RR: Relative Risks
SMRs: Standardized Mortality Ratios
