# Supplementary material for: Small-area socioeconomic deprivation indices in Cyprus: development and association with premature mortality
Source: BMC Public Health. 2019 May 22;19:627. doi: 10.1186/s12889-019-6973-0 (PMC6532164; doi:10.1186/s12889-019-6973-0)
Supplement: Supplementary file 1 — Median values of the census indicators for each deprivation index and quartile. Summary measures of the census indicators for each deprivation index and quartile (DOCX 12 kb) [file 12889_2019_6973_MOESM1_ESM.docx]

**Table 1.** Median values of the census indicators for each deprivation index and quartile

| **Index** | **Census indicator** | **Q1-least**  **deprived**  **(n=92)** | **Q2**  **(n=93)** | **Q3**  **(n=91)** | **Q4-most**  **deprived**  **(n=93)** |
| --- | --- | --- | --- | --- | --- |
| **Rural-related**  **SE deprivation**  (n=369 municipalities/  communities) | Population with low  education level | 36.9% | 43.4% | 55% | 68.5% |
|  | Single person households | 14.2% | 17.8% | 21.7% | 28.1% |
|  | Divorced or widowed population | 6.5% | 8.1% | 11.1% | 18.5% |
|  | Single parent household | 7.1% | 5.7% | 4.9% | 3.6% |
| **Material deprivation**  (n=369 municipalities/  communities) | Unemployed economically active  population | 8.7% | 9.2% | 10.1% | 13.6% |
|  | Not owner occupied households | 9.6% | 18.6% | 26.3% | 41.2% |
|  |  | **Q1-least**  **deprived**  **(n=30)** | **Q2**  **(n=30)** | **Q3**  **(n=29)** | **Q4-most**  **deprived**  **(n=30)** |
| **Urban-specific**  **SE deprivation**  (n=119 municipalities/  Communities) | Unemployed economically active  population | 9.3% | 9.5% | 10.1% | 12.8% |
|  | Not owner occupied households | 17.2% | 18.6% | 26.4% | 41.8% |
|  | Divorced or widowed population | 5.6% | 6.5% | 7.6% | 9.1% |
|  | Single parent household | 5.7% | 7.0% | 7.3% | 8.6% |
